# Agreement between Ventilatory Thresholds and Bilaterally Measured Vastus Lateralis Muscle Oxygen Saturation Breakpoints in Trained Cyclists: Effects of Age and Performance

**DOI:** 10.3390/sports12020040

**Published:** 2024-01-28

**Authors:** Karmen Reinpõld, Indrek Rannama, Kristjan Port

**Affiliations:** School of Natural Sciences and Health, University of Tallinn, 10120 Tallinn, Estonia; rannama@tlu.ee (I.R.); kport@tlu.ee (K.P.)

**Keywords:** NIRS, SmO_2_ breakpoints, gas exchange, ventilatory thresholds, high-level cycling, performance, phenotype

## Abstract

This study focused on comparing metabolic thresholds derived from local muscle oxygen saturation (SmO_2_) signals, obtained using near-infrared spectroscopy (NIRS), with global pulmonary ventilation rates measured at the mouth. It was conducted among various Age Groups within a well-trained cyclist population. Additionally, the study examined how cycling performance characteristics impact the discrepancies between ventilatory thresholds (VTs) and SmO_2_ breakpoints (BPs). Methods: Junior (n = 18) and Senior (n = 15) cyclists underwent incremental cycling tests to assess their aerobic performance and to determine aerobic (AeT) and anaerobic (AnT) threshold characteristics through pulmonary gas exchange and changes in linearity of the vastus lateralis (VL) muscle SmO_2_ signals. We compared the relative power (Pkg) at ventilatory thresholds (VTs) and breakpoints (BPs) for the nondominant (ND), dominant (DO), and bilaterally averaged (Avr) SmO_2_ during the agreement analysis. Additionally, a 30 s sprint test was performed to estimate anaerobic performance capabilities and to assess the cyclists’ phenotype, defined as the ratio of P@VT2 to the highest 5 s sprint power. Results: The P_kg_@BP for Avr SmO_2_ had higher agreement with VT values than ND and DO. Avr SmO_2_ P_kg_@BP1 was lower (*p* < 0.05) than P_kg_@VT1 (mean bias: 0.12 ± 0.29 W/kg; Limits of Agreement (LOA): −0.45 to 0.68 W/kg; R^2^ = 0.72) and mainly among Seniors (0.21 ± 0.22 W/kg; LOA: −0.22 to 0.63 W/kg); there was no difference (*p* > 0.05) between Avr P_kg_@BP2 and P_kg_@VT2 (0.03 ± 0.22 W/kg; LOA: −0.40 to 0.45 W/kg; R^2^ = 0.86). The bias between two methods correlated significantly with the phenotype (r = −0.385 and r = −0.515 for AeT and AnT, respectively). Conclusions: Two breakpoints can be defined in the NIRS-captured SmO_2_ signal of VL, but the agreement between the two methods at the individual level was too low for interchangeable usage of those methods in the practical training process. Older cyclists generally exhibited earlier thresholds in muscle oxygenation signals compared to systemic responses, unlike younger cyclists who showed greater variability and no significant differences in this regard in bias values between the two threshold evaluation methods with no significant difference between methods. More sprinter-type cyclists tended to have systemic VT thresholds earlier than local NIRS-derived thresholds than athletes with relatively higher aerobic abilities.

## 1. Introduction

Exercising at a metabolically optimal intensity is an essential precursor for achieving high performance [1]. Until now, when assessing aerobic (AeT) and anaerobic (AnT) thresholds, a pulmonary gas exchange analysis was considered as a golden standard [2], although alternative methods are available. One of these is near-infrared spectroscopy (NIRS), which measures muscle oxygenation in the working muscle [3,4]. In the past decade, the field of performance development has been increasingly data-driven; therefore, the ability to measure performance-related parameters on the field is necessary.

Evaluating thresholds through a pulmonary gas exchange analysis or blood lactate level measurements indicates the overall metabolic response rate of the entire body [5]. Despite their connection to skeletal muscle metabolism, these methods are unable to distinguish the contributions of various muscle groups (s.c., peripheral components) [6]. However, when the focus is on long-term aerobic endurance and fatigue tolerance, then the main limiter is not so much about the systemic contribution (VO_2max_) as it is the local component of the body [7], predominantly the muscle’s ability to sustain the effort and fight the fatigue [8]. Surface electromyography (EMG) has traditionally been utilised to understand the local component in laboratory conditions. It is considered relatively low-cost and effective, although a post-measurement data analysis can be highly time-consuming; therefore, practitioners rarely use it [9].

The wearable industry is stepping rapidly forward, and one of the applications, among other performance-related technologies, is portable NIRS [3]. Understanding the muscle’s ability to consume oxygen can give insight into the metabolic processes that help or limit the athlete’s performance [4,10]. A relatively high number of studies have been performed in past years to assess the NIRS ability to evaluate metabolic thresholds [11,12,13], and the overall consensus supports that in the NIRS signal, there are distinctive breakpoints (BPs), which are coinciding with different thresholds—ventilatory, lactate, gas exchange, etc.

Nevertheless, the general practice for study design has been comparing oxygen uptake data to muscle deoxygenation [14,15,16]. Although, commonly available NIRS devices measure oxygen saturation in arbitrary units and therefore it would be more applicable to use the same parameters the data are measured in as has been performed in latter studies [11,12,13,17,18]. As mentioned before, many studies have been performed to establish the correlation between thresholds identified using pulmonary gas exchange devices and NIRS signals. However, there is a lack of consensus on the methods used for identifying thresholds from NIRS signals. At the same time, a considerable number of studies are identifying only either first [19] or second NIRS BP [14,16,20]. Moreover, some studies claim to assess the first BP from the NIRS signal [17,21], although this BP seems not to be driven by the qualitative changes in metabolic processes but rather arising from the initial reactions of adapting muscle (increased blood flow) to increased intensity [19]; therefore, the emerging time of the first breakpoint is often underestimated.

Most research in this field focuses on recreational populations, characterized by high variability in various parameters. This highlights the necessity for research involving more uniform groups, such as elite or well-trained endurance athletes, which would be particularly useful for practitioners like coaches. Additionally, a comparative analysis between these two groups would be beneficial in its own right. We know the maximal oxygen uptake is relatively highly developed by the twenties age range, and from thereon, there is no considerable rise in absolute values from the level established before [22]. Therefore, the global component may well be founded early in the endurance athlete’s career; nevertheless, the local component may be the limiter of the performance. On the contrary, well-trained endurance athletes possess years of experience and specialized training, which may contribute to a higher local muscle ability to utilize oxygen. This increased efficiency could potentially be greater compared to younger athletes, even when the maximal oxygen uptake may be in decline [23,24]. Near-infrared spectroscopy is claimed to directly measure the oxygenation of haemoglobin (Hb) in the capillary bed of surface muscles (mainly in capillaries) and myoglobin (Mb) in the muscle cytoplasm, indicating non-invasively skeletal muscle oxidative metabolism in vivo during exercise [25] and is reflecting, therefore, local oxygen metabolism. Measuring oxygen uptake through gas exchange parameters represents global O_2_ consumption. Thus, the following question arises: are the thresholds for younger and older athletes measured with global and local components different because of their training heritage and age difference using the NIRS method?

Another aspect to consider is a so-called phenotype of endurance athletes driven by the muscle fibre type prevalence, i.e., time-trailer or climber-type cyclist. They have predominantly slow-twitch muscle fibres in the working muscle and an all-rounder or sprinter-type has a higher percentage of fast-twitch muscle fibres [26]. Because of the working muscle’s structural and quantitative difference, the sprinters have higher maximal power and a relatively low anaerobic threshold compared with time-trailer or climber-type cyclists [27]. Therefore, this may also influence the local and global oxygen dynamics when assessing thresholds. A current study may add more insight into the between-subject variability and correspondence between threshold values assessed using NIRS and pulmonary gas exchange methods [4].

Measuring the local oxygen utilisation with the NIRS system takes into account only one or a few independent muscles, which is therefore considered a limitation of this system. Similarly, it is found that NIRS measurements have remarkable intermuscular heterogeneity in O_2_ saturation (SmO_2_) kinetics [28], which reduces after priming exercise [29]; however, less is known about bilateral homogeneity of the same muscle group and region during a workload increase on bilaterally equal movements such as cycling. The remarkable bilateral asymmetries in the pedalling kinetics, kinematics, and muscular activity characteristics have been documented in the research on cycling biomechanics [30]. Some evidence exists that pedalling force-generating patterns may differ between dominant and non-dominant legs [31]. At the same time, studies are scarce comparing muscle groups bilaterally [32], often using either the dominant leg or right leg with no apparent reason, or even though both limbs are measured, the results are not represented in outcomes [12,33]. Furthermore, the pedalling pattern may be related to athletes’ phenotype. Therefore, there may be some differences in oxygen saturation kinetics between the left and right leg. Moreover, aggregating the signal of the main working muscles on both body sides may relate the local and global oxygen kinetics and the metabolic thresholds more strongly [32].

Previous research [34] suggests that while systemic and NIRS-based local threshold values generally align at the group level, they show significant individual variability that could stem from methodological biases or biological factors. Understanding the reasons behind these individual differences is crucial for managing practical training processes. Therefore, practitioners need to be aware of the potential characteristics that differentiate systemic and local thresholds. 

Therefore, this study aimed to analyse the agreement between metabolic thresholds determined from physiological signals of bilaterally measured local muscle oxygen saturation using NIRS and global pulmonary ventilation measured at the mouth in the context of different Age Groups among a well-trained cyclist population and also analyse the effect of cycling-performance-related characteristics, comparing ventilator thresholds and muscle oxygen saturation breakpoints.

## 2. Materials and Methods

### 2.1. Participants

Fifteen Senior (Seniors)-class, trained or well-trained [35] male cyclists and eighteen young male Junior- and U23-class (grouped as Juniors) national- and international-level cyclists took part in this study (detailed description in Table 1). All Seniors had at least 10 (between 11 and 31) years of continuous endurance cycling training experience with more than 5000 (6200–14,900 km during past season) km per year. None of them had a professional cycling background, and all had sporting history from childhood, but most of them started cycling as a performance-oriented sport in adulthood. Juniors had at least 4 (4 to 8) years of continuous endurance cycling training (>5000 km/year) experience with more than 10,000 km (10,200–25,300 km) during the past season. Study participants were recruited through the Estonian Cycling Federation and coaches of national teams among licenced U19, U23, and Senior (up to 50–54 age categories) road cyclists. All cyclists who did not meet the described training-history-related criteria or had health issues were excluded from the study. Except for one Junior participant, the dominant leg, determined by ball-kicking preference, was the right leg for all participants. All athletes had previous experiences with laboratory testing procedures. The Tallinn Medical Research Ethics Committee approved the study in compliance with the Declaration of Helsinki. All participants provided written informed consent.

### 2.2. Study Design

A between- and within-subjects cross-sectional design was used to compare two different methods for evaluating two breakpoints on dynamics of different physiological measures during incremental exercise, which generally are defined as aerobic (AeT) and anaerobic (AnT) thresholds. All experimental procedures per participant were made during one day during the early part (February to March) of the cycling competition period. Participants were asked to maintain a regular, well-balanced diet, avoid strenuous exercise for at least 48 h before the laboratory test, and abstain from caffeine on the day of testing. The incremental cycling exercise and 30s sprint test on the cyclists’ bike were performed to measure cycling-specific performance and physiological reactions to the external load. Ventilator thresholds (VT1 and VT2), expressing global reactions to a workload increase, were evaluated with gas exchange characteristics, and linearity changes in the local muscle level were determined as breakpoints (BP1 and BP2) in *vastus lateralis* muscle oxygen saturation (SmO_2_) signals in the non-dominant (ND) and dominant (DO) leg. The intra-subject comparison between power values measured at VT1 and BP1 and VT2 and BP2 was made, and inter-subject agreement between power levels of two different methods was evaluated. Additionally, the effect of different anthropometrics, training statuses, and performance characteristics on differences between VT1 and BP1 and VT2 and BP2 power values were evaluated.

### 2.3. Procedures and Measurements

*Cyclists’ training-history*-related questionnaires were conducted after the arrival at the laboratory, and questions about training record (continuous years of cycling training at 5000 km per year at least), seasonal cycling distance during the past full year, and training aims were asked. 

*Participants’ anthropometrical characteristics* (height and body mass) were measured after arrival at the laboratory with a Seca mBCA 514/515 body composition analyser (Medical Measurement Systems and Scales, Hamburg, Germany). Double skinfold thickness was measured with a Baseline Skinfold Caliper (Fabrication Enterprises, Inc., Elmsford, NY, USA) at the site of application of the NIRS sensor, and this measurement was divided by 2 to obtain the adipose tissue thickness (ATT) [36]. The ND and DO leg values were averaged and used as a single ATT measurement for every participant.

*For determination of the ventilatory thresholds, muscle oxygen saturation BP, and cycling performance characteristics*, the athletes underwent two different cycling tests on their personal road bikes, which were mounted on a Cyclus2 cycle ergometer (RBM Elektronik-Automation, Leipzig, Germany). This setup was considered both valid and reliable based on Rodger’s study [37]. 

*The experimental testing procedure* included an incremental cycling test to evaluate the cyclist’s aerobic performance characteristics and a 30 s maximal sprint test to determine anaerobic performance abilities. The 20 min of active recovery, which included a 10 min ride with a power of 100 W and a 10 min ride with an intensity equal to ~80% of power measured at VT1, was enabled between the end of incremental exercise and the start of the sprint test. 

*The incremental test protocol* began with a 7 min warm up at 90 W. The baseline power started from 100 W and increased by 30 W every 3 min. The athletes maintained the same seated cycling position throughout the test, hands on brake hoods and pedalling cadence between 90 and 100 rpm. 

*Respiratory and pulmonary gas exchange variables* were measured using a breath-by-breath gas analyser (Quark PFTergo, Cosmed, Rome, Italy). The gas analyser was calibrated before each test using reference gases (5% CO_2_; 16% O_2_) and a known volume syringe (3 L), following the manufacturer’s instructions.

*The continuous recording of oxygen saturation* (SmO_2_) in the ND and DO vastus lateralis (VL) muscle was performed using the mobile NIRS device Moxy Monitor (Fortiori Design LLC, Hutchinson, MN, USA), established as valid and reliable [11,33]. A Moxy Monitor measures the amount of NIR light reaching from one emitter to two detectors (12.5 and 25 mm from the emitter) at four wavelengths (between 630 and 850 nm), with an adjustable output sampling rate between 0.5 and 2 Hz, and uses the Monte Carlo model for signal processing, which is described in more detail elsewhere [33]. The Moxy Monitor probes were symmetrically placed on the ND and DO leg VL muscle, approximately two-thirds of the distance between the anterior superior iliac spine and the lateral side of the patella [33]. Prior to placing the probes, any body hair in the area was removed, and the sensors were covered with a compatible light shield available on the market. The sensors were secured in place using medical adhesive tape to ensure their stability. The Moxy Monitor was connected with the Garmin Edge 520 (Garmin, Olathe, KS, USA) head unit using the ANT+ signal protocol, and data were captured with 1Hz. Also, the Favero Assioma Duo pedal power meter (Favero Electronics, srl., Arcade, TV, Italy) was connected to the head unit to provide additional power reference data for the NIRS signal. The Cyclus2 ergometer was controlled using gas analyser software Cosmed OMNIA 2.1 (Cosmed, Rome, Italy), and the gas analyser and Garmin Edge head unit data collection was synchronised. 

*The 30 s sprint cycling test was conducted in the seated position, with hand brakes contributing to drops in the isokinetic mode with a fixed cadence of 110 rpm*. During the sprint test, the cyclists were instructed to start as powerfully as they could and to hold maximal effort until the end of the test. The riders were verbally encouraged to exert their maximum effort during the whole sprint test and at the end of the incremental test.

### 2.4. Computations and Measurements

The gas analyser and muscle SmO_2_ signals were integrated into 10 s time-bins to reduce the signal-to-noise ratio before threshold and BP analyses were conducted. 

*The ventilatory threshold analyses* were performed with Cosmed OMNIA 2.1 (Cosmed, Rome, Italy) software independently by two physiologists, and a third one was consulted in case of disagreement. The first ventilatory threshold (VT1) related to the aerobic threshold was determined as the first breakpoint of the VCO_2_/VO_2_ vs. power. The second ventilatory threshold (VT2) related to the anaerobic threshold was identified based on (a) a second slope increase on the curve between minute ventilation (VE) and power, (b) a second increase in the ventilatory equivalent for oxygen and ventilatory equivalent for carbon dioxide, and (c) a decrease in end-tidal partial pressure of carbon dioxide, as outlined by Wasserman [38]. The highest 30 s mean value of VO_2_ was recorded as the VO_2max_. The average 30s VO_2_ value around the time point of ventilator thresholds was used as VO_2_ characteristics at VT1 (VO_2_@VT1) and VT2 (VO_2_@VT2). Power values for Peak Power (PP), VT1 (P@VT1), and VT2 (P@VT2) were calculated proportionally using the time when the respective criteria were achieved (P [W] = P of last completed workload [W] + 30 [W] × Time on last uncompleted workload [s]/180 [s]). The absolute and relative (normalised with body mass) threshold and peak values of P (P_kg_) and VO_2_ (VO_2kg_) were used as indicators of the athlete’s aerobic performance. 

*The anaerobic power and capacity* were described with absolute (W) and relative (W/kg) power of the highest 5s (Pmax5s and P_kg_max5s) and average 30s sprint power (Pmax30s and P_kg_max30s) captured during the sprint test. The athlete’s typology in the current study is handled as an intrapersonal relative balance between sprinting and aerobic endurance capabilities and is quantitatively expressed as the ratio between Pmax5s and P@VT2.

**Figure 1:**
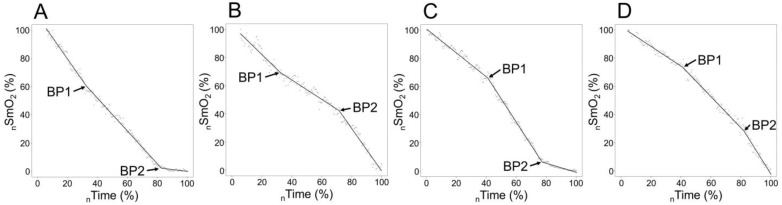
Four different types of signal slope change combinations and breakpoints, where plot (**C**) represents typical sloping pattern and (**A**,**B**,**D**) are atypical; the scales of axes are normalised for individual ranges in SmO_2_ (_n_SmO_2_) detected during the test (Min SmO_2_ to Max SmO_2_) and for incremental test duration (start of the first step to the end of exercise) to time (_n_Time) scale. First breakpoint (BP1) and second breakpoint (BP2).

*The signal processing and BP analyses for SmO_2_ signals* were performed in an R-programming environment. The automated segmented (piecewise) regression analysis with the package “segmented” [39] with a maximum of two breakpoints and a minimum segment length of 180 s was performed to detect BP. The minimal sum of square residuals was used as criteria for the best fit of the function. According to the general tendency for SmO_2_ values to initial increase during the start of exercise [19], the BP modelling starting point was set at the time when the maximum SmO_2_ value was achieved, and the signal started to systematically decrease (usually after the first increment at 100 W). The endpoint for modelling was set at the end of the exercise when the athlete could not maintain the predefined cadence. The first change point in the SmO_2_ signal slope was defined as BP1 and the second change as BP2. If only one PB was found (n = 1) in the signal, then, if it happened during the first 70% of the incremental test, it was handled as BP1 (n = 0); if later, then it was handled as BP2 (n = 1). According to slope change directions (reduction or increase in SmO_2_ change rate in time) after two BP, theoretically, four different types of sloping combinations may be presented, which are visually illustrated in Figure 1. The typology of SmO_2_ signal sloping was registered for each participant and both body sides.

Similarly, with P@VT1 and P@VT2, the power values at BP1 (P@BP1) and BP2 (P@BP2) for the ND and DO leg were computed proportionally from the time points when those BPs were detected. Additionally, for the ND and DO side P@BP values, the averaged power value for BP1 (P@BP1 of Avr SmO_2_) and BP2 (P@BP2 of Avr SmO_2_) of both legs was computed. P values at the aerobic and anaerobic level, either from ventilation or SmO_2_ signals, were computed from the time when level determination criteria were met, and therefore lower P values mean an earlier occurrence of a certain intensity level with 1W equal to 6 s.

To compare absolute saturation level values between Age Groups, the highest (Max SmO_2_) and lowest (MinSmO_2_) 10s average SmO_2_ values, measured during the initial and final part (accordingly) of the incremental test, for ND and DO sides, were incorporated into a future analysis, as well as the modelled SmO_2_ values at BP for the ND and DO leg.

### 2.5. Statistical Analysis

The descriptive statistics for measured characteristics are presented as the mean and standard deviation (SD), and the relative variance of measurements is described using the coefficient of variation (CoV). The assumption for data normality was controlled with the Shapiro–Wilk normality test, and for Age Group comparisons, the equality of variances was tested with Levene’s test. The differences between Age Group characteristics were controlled with Student *t*-tests for independent samples or with the Mann–Whitney test in cases where assumptions for parametric tests were not met. The Student *t*-test for paired samples was used to control the differences between ND and DO leg SmO_2_ characteristics. As a common practice variable for cycling training and performance monitoring, the relative power (P_kg_) value (W/kg) was used to compare corresponding VT and SmO_2_ BP-based intensity levels. The ANOVA for repeated measures was used to control the significance of differences between P_kg_ values at VT and SmO_2_ BP (for DO, ND, and average measures separately) as well as between P_kg_ at the ND and DO side SmO_2_; the Age Groups as between-participant variables were used to control the effect of long-term cycling training experience to named differences in power level values. The Bonferroni post hoc test was applied if the general ANOVA model indicated significant differences.

To evaluate the agreement level between corresponding VT and BP and between ND and DO SmO_2_-related P_kg_ values, the Bland Altman plots with upper and lower Limits of Agreement (LOA) at a 1.96 SD level were established. Bland Altman analyses were performed on the whole sample and for separate Age Groups to evaluate possible age-related differences in agreement between local and systemic threshold values. To evaluate agreement in covariation between VT and BP and between ND and DO SmO_2_-related power values, a simple regression analysis and plots were conducted, and the coefficient of determination (R^2^) was used to measure the strength of associations. 

Correlation and partial correlation analyses were performed to control the effect of cycling-specific performance characteristics on a mismatch between corresponding VT and BP power values (bias). For the interpretation of correlation coefficients, the threshold levels 0.1, 0.3, 0.5, 0.7, and 0.9 for small, moderate, large, very large, and extremely large were used [40].

To control statistical significance between Age Group distribution in SmO_2_ signal sloping types, the Chi-Squared test was used. 

Statistical analyses were performed using JASP 0.18.1 computer software (JASP Team, 2023). The level of statistical significance was set at 95% (*p* < 0.05). Additionally, the effect size of Cohen d > 0.2 was used to confirm the statistical difference in the *t*-test results. Interpreting the effect size is as follows: 0.2 = small effect; 0.5 = moderate effect; 0.8 = large effect [40].

## 3. Results

### 3.1. Performance Characteristics

Table 2 presents the characteristics of aerobic and anaerobic performance for Junior and Senior cyclists. Junior cyclists possess remarkably (*p* < 0.05; d > 1) higher relative aerobic and anaerobic performance values than Seniors, and their typology was more directed to the sprinting side. In absolute performance abilities, Juniors also had significantly higher sprint test results (large effect size) and power values related to VT1 and VT2 thresholds and PP (moderate effect size), but no between-group differences existed in absolute VO_2_ values at different intensities. 

### 3.2. Typology and Breakpoints of SmO_2_ signal sloping according to Age Group

Table 3 presents minimum, maximum, and BP-related SmO_2_ values in the original scale (0–100%), and in general, no significant differences were found between Juniors and Seniors. Similarly, no significant (*p* > 0.05) differences existed between ND and DO leg values in the whole sample or separate Age Groups. 

In our data, we could detect four different types of signal change when accounting for BP, which are visualised in Figure 1. The most common one is on chart C, represented in 55 cases out of 66 (two body sides for 33 participants). Type A occurred in five, B in two, and D in four cases. The bilateral combination of sloping types was as follows: C–C, n = 24; C–D, n = 3; C–B, n = 2; C–A, n = 2; A–A, n = 1; A–D, n = 1. Out of 11 cases with no C–C combination represented, 5 were Seniors, and 4 were Juniors. The classical C type did not occur in five cases on the ND side and six on the DO side. One Junior cyclist had no identifiable BP1 on the ND side, but BP2 was typical for the C type. No statistically significant differences existed in slope type distribution between Age Groups in the ND (*p* = 0.254) and DO leg (*p* = 0.567).

**Figure 2:**
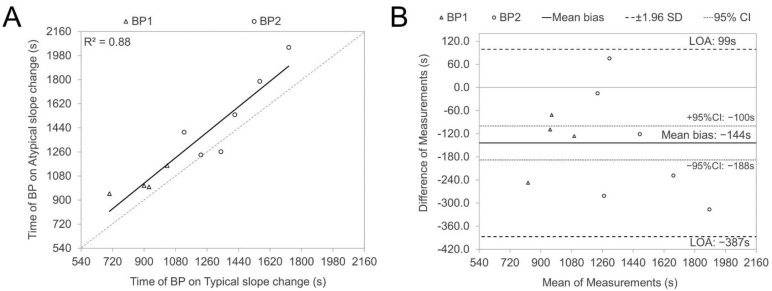
Regression and Bland Altman plots were used to illustrate the relationships (**A**) and correspondence (**B**) between the timing of SmO_2_ signal breakpoints (BPs) for typical (types C and D for BP1; A and C for BP2) and atypical (types A and B for BP1; B and D for BP2) slope changes. These plots were particularly focused on instances where opposing directions of slope changes were observed in the contralateral VL muscles, with the difference being calculated as typical minus atypical.

The atypical SmO_2_ signal sloping illustrated in plots A, B, and D in Figure 1 was, in most cases, presented in one leg in combination with the C type on the contralateral side (seven cases out of nine) for the same subject and only for one participant, type A was presented bilaterally. To control the timing agreement between typical and atypical slope changes, we compared the time points of BP1 and BP2 occurrences between typical slope changes, presented as type C in Figure 1 (as well as type D for BP1 and A for BP2), and atypical, reverse directional (types A and B for BP1; B and D for BP2) changes. Figure 2 (plot A) illustrates a strong correlation (r = 0.94, *p* < 0.05) between BP time points of typical and atypical sloping of contralateral legs, but the Bland–Altman analysis (plot B) revealed low agreement between contralateral BP timing values. The occurrence of BP on typical slope changes is in almost all cases (9 of 10) earlier than BP on the contralateral (atypical) side, presented as a significant (*p* < 0.05) 144s average time difference.

Table 4 represents the power values for two SmO_2_ BPs for the non-dominant and dominant sides and the average values of those two signals. Similar to absolute P@VT characteristics but with large effect sizes, the Juniors’ P@BP values are significantly higher than the Seniors’ power values, except for the BP2 for the ND leg.

### 3.3. Agreement between Ventilatory Thresholds and Bilaterally Measured SmO_2_ Breakpoints and between ND- and DO-Side SmO_2_ Breakpoints among Different Age Groups

#### 3.3.1. Agreement between VT1 and BP1

**Figure 3:**
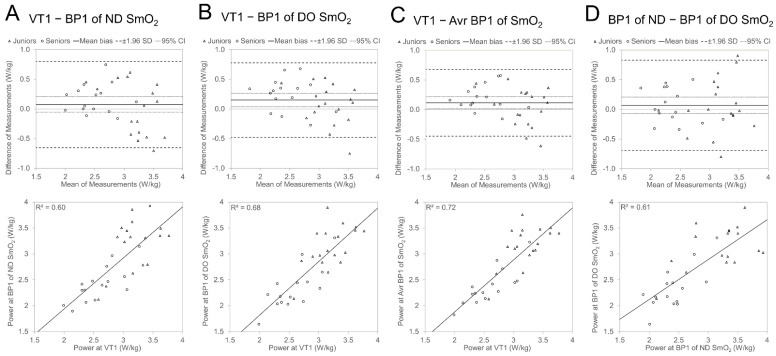
Bland Altman and regression plots between relative power (W/kg) values of VT1 and originally measured SmO_2_ BP1 of ND side (section **A**) and DO side (**B**); average value of ND and DO side (**C**) and relationship between power values of ND- and DO-side SmO_2_ BP1 (**D**).

Figure 3 visualises through Bland Altman and regression plots the agreement between VT1- and BP1-related power values for the ND and DO legs as well as the averaged value of the ND and DO leg. The individual values of participants from different Age Groups are presented with different symbols, and averaged Age Group characteristics of agreements are presented in Table 5. The Bland–Altman analysis presented a 0.08 ± 0.37 W/kg average difference (mean bias) between P_kg_@VT1 and P_kg_@BP1 for the ND side, and it was statistically not different from zero (Figure 2, panel A), but 95% of the differences in power between the two methods (LOA) were expected to fall in the range of −0.65 to 0.81 W/kg. For the DO side, the difference was 0.15 ± 0.32 W/kg, and the P@BP1 value was significantly (*p* < 0.05) lower than the P@VT1 value (panel 2B), and LOA (−0.48 to 0.78 W/kg) was slightly narrower than that found to be in the ND leg. The LOA (−0.45 to 0.68 W/kg) of the averaged P@BP1 value was even smaller than for the ND or DO leg separately, and the mean bias (0.12 ± 0.29 W/kg) was significantly different from zero. The regression analyses presented significant (*p* < 0.05) relationships between power values at VT1 and BP1. However, the level of covariation between P@VT1 and P@BP1 was highest for the DO side compared to ND (lower plots in Figure 3 in panels A and B), and the determination coefficient for the averaged combined (panel 2C) value was higher than for both legs separately. 

The mean difference in P@BP1 between ND and DO sides was 0.07 ± 0.39 W/kg, and it was statistically not different from zero (Figure 3, panel D), but LOA was relatively wide (−0.69 to 0.83 W/kg). The description level of covariation between leg P@BP1 values was slightly higher than 60%. 

The comparison of differences/agreement between ventilatory thresholds and SmO_2_ breakpoints between Age Groups (Table 5) showed a tendency for lower BP1 power values against VT1 (earlier occurrence of BP1 against VT1) for Senior cyclists compared with Juniors. However, it did not show any statistical significance. When the agreement of the two methods was analysed in separate Age Groups, in all cases, the P_kg_@BP1 was significantly (*p* < 0.05) lower than P_kg_@VT1 in the Seniors group, but in the Juniors group, the differences in P_kg_ values between the two methods were not different from zero. The LOA was in all cases more than 0.25 W/kg wider in the Juniors group than in the Seniors group.

#### 3.3.2. Agreement between VT2 and BP2

**Figure 4:**
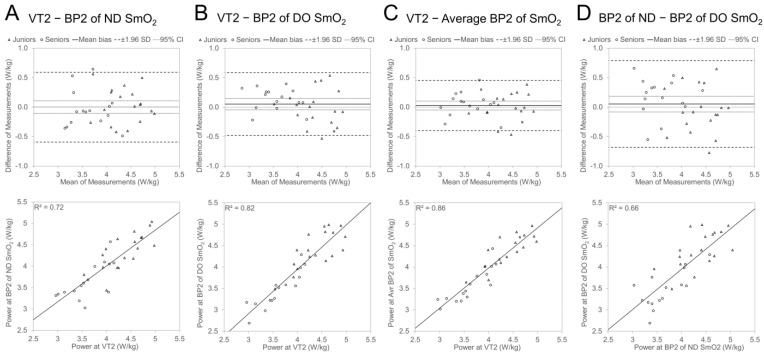
Bland Altman and regression plots between power values of VT2 and SmO_2_ BP2 of ND side (**A**) and DO side (**B**); average value of ND and DO side (**C**) and relationship between power values of ND- and DO-side SmO_2_ BP2 (**D**).

The agreements between AnT-related power values are illustrated in Figure 4. The mean bias between P_kg_@VT2 and P_kg_@BP2 for the ND side (mean bias: 0 ± 0.30 W/kg; LOA: −0.60 to 0.59 W/kg), DO side (0.05 ± 0.27 W/kg; LOA: −0.48 to 0.58 W/kg), and averaged value (0.03 ± 0.22 W/kg; LOA: −0.40 to 0.45 W/kg), nor between P_kg_@BP2 values of the ND and DO side (0.05 ± 0.38 W/kg; LOA: −0.68 to 0.79 W/kg), was statistically not different from zero. Similar to the AeT, the determination coefficient was higher, and LOA was narrower between P_kg_@VT2 and P_kg_@BP2 for the DO side compared with the same result for the ND side and the averaged value of the ND and DO leg presented higher agreement than both sides separately.

Compared to the AeT level, the agreement and covariation between power values at VT2 and BP2 were higher and different from the AeT, where P_kg_@BP1 tended to be lower than P_kg_@VT1; there was no clear tendency for the differences between BP2 and VT2 values.

The mean bias values between P_kg_@VT2 and different P_kg_@BP2 characteristics were not statistically (*p* > 0.05) different between Age Groups (Table 6). The agreement analyses in separate Age Groups mainly did not show significant differences between the P_kg_ values of the two methods, except in one characteristic in the Seniors group where P_kg_@BP2 of the DO leg was significantly (*p* < 0.05) lower than P_kg_@VT2. 

### 3.4. Effect of Cyclist’s Performance Characteristics on Differences between Ventilatory Thresholds and SmO_2_ Breakpoints

Table 7 presents the Pearson and partial correlation analysis results between cyclists’ performance characteristics and global and local threshold value differences in separate Age Groups and in the whole sample. The Age Group was used as a controlling variable when all participants were analysed together because of the significant differences in performance characteristics between groups. One Junior participant was eliminated from the AeT-related analysis as an outlier (a more than two SD difference from the group average in bias value between the two methods). 

**Figure 5:**
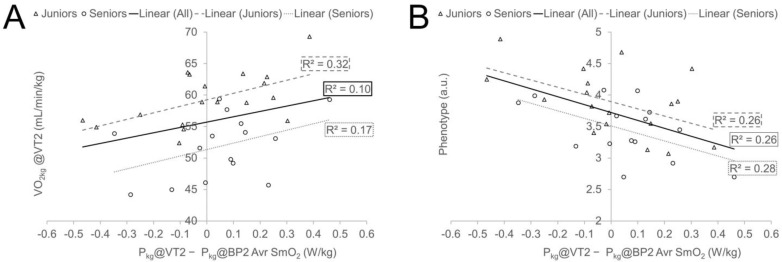
Regression plots representing associations between selected cycling performance characteristics (plot (**A**) relative VO2@VT2 and plot (**B**) cyclists’ typology) and bias value between relative power at VT2 and BP2 of SmO_2_ (average of ND and DO leg).

The correlation results (Table 7) indicate that individuals with higher relative VO_2kg_ levels at VT2 tend to reach their AnT at lower power values (more early) at the local (i.e., NIRS) level than globally measured VT2 (illustrated graphically in Figure 5, panel A). On the other hand, phenotypically, more sprinter-like athletes (higher P5s/P@VT2 ratio) encountered either a VT2 earlier before reaching BP2 locally in a muscle SmO_2_ or the BP2 was to a lesser extent earlier than VT2. Similar trends existed in both Age Groups separately and in the whole sample if the Age Group effect was eliminated (Figure 5, panel B). The significant (*p* < 0.05) effect of the phenotype to bias between VT1 and BP1 was also confirmed in the whole sample, especially in the Seniors group, but not in the Juniors group separately. No other linear relationships were found between VT1 and BP1 bias and performance characteristics. 

Analogous to performance characteristics, we also controlled the effect of anthropometric (height, body mass, BMI, and ATT) and training-history-related (years of cycling training and km covered during past complete season) characteristics on the P_kg_ bias value between the two methods and did not find any significant (*p* > 0.05, −0.30 < r < 0.30) linear relationships between said variables.

## 4. Discussion

The present study was focused on two main objectives: (1) to examine the concordance between metabolic thresholds derived from bilaterally measured local muscle oxygen saturation signals using NIRS and global pulmonary ventilation rates measured at the mouth, across various age groups in a population of well-trained cyclists, and (2) to explore how characteristics related to cycling performance might influence discrepancies between ventilatory thresholds and muscle oxygen saturation breakpoints. 

### 4.1. Breakpoint Evaluation and Signal Sloping

The methodology for determining ventilatory thresholds from pulmonary ventilation characteristics is well defined and considered one of the gold-standard methods in sports physiology. At the same time, the methods for evaluating the metabolic thresholds from the local muscular level from NIRS signals are not so clear, and different methods have been proposed [34]. In the current study, we used the piecewise regression technique as a principally used method [34], but contrarily to many previous studies, we used the SmO_2_ signal instead of the often used HHb value [14,15,16]; we started modelling after the initial warm-up-related rise in SmO_2_ dynamics, which is sometimes handled as the first BP [17,21]; analysed two BPs instead of one BP [12,19,21,41]; and accounted for both legs on BP analyses with the aggregation (averaging) of contralateral values, to understand the agreement between contralateral values and the possible timing effect between local and global threshold values. 

As a result of the identification of two breakpoints, we came across four distinct signal combinations, as illustrated in Figure 1; two BPs were found from 65 cases out of 66 (both legs for 33 participants) and from one body side for one participant, and only BP2 was determined. The most typical one, which is established in most of the studies conducted on this topic in cycling [13,21], was type C, where the signal would start with a gentle downward slope; after the first BP, it gets considerably steeper and thereafter, when accounting for the second BP, the signal levels off again. 

Generally, the physiological appearance of the first BP is primarily due to increased glucose oxidation, and lactic acid build-up triggers the activation of acid-sensing ion channels [42]. At the muscular level, it is expressed as an increased O_2_ consumption rate per unit of work that can be measured as an increased rate of the deoxygenation value in the NIRS signal. This local activation corresponds to an increase in signals sent to the cardiovascular and respiratory centres, resulting in heightened blood pressure, heart rate, left ventricular contractility, and ventilation [43] as markers of VT1. The physiological events occurring at the AnT are complex and multifactorial [44,45], but at the muscular level, it may indicate the exhaustion of the potential of aerobic processes mainly because of an imbalance between O_2_ supply and utilisation [19]. The O_2_ supply to muscle is related to the capillary contact area in muscle [5], and insufficient O_2_ supply to muscle cells may be related to the fulfilment of the potential of oxygen diffusion capacity or with (mechanical) restrictions to use this potential. It has been hypothesised that high intramuscular tension due to an increased workload reduces the blood flow to the muscle and inhibits local work capacity [19,46]. Those interpretations match well with type-C sloping, which was found to be more common in our study and described by most previous studies analysing NIRS signal dynamics during incremental or ramp-type cycling exercises [15]. At least two previous studies [17,47] have illustrated VL SmO_2_ signal sloping during a continuous work rate increase, where after both BPs, the signal downward slope got steeper, illustrated as type D in Figure 1 in our study, but both of those studies were conducted during incremental running exercises and in one of those studies 30s rest periods between workloads were allowed and sloping of SmO_2_ change rates per workload was studied [47]. Similar sloping patterns have been described for non-primary working muscle during cycling incremental exercise [12]. 

One additional finding of our study revealed that atypical SmO_2_ signal sloping (as shown in plots A, B, and D of Figure 1) occurred predominantly (in seven out of nine cases) in one leg, paired with a classic type (depicted in plot C of Figure 1) in the opposite leg, with a notably later BP timing (as seen in Figure 2, plot B). These delayed atypical slope changes might represent compensatory responses due to certain bilateral asymmetries (be it biomechanical, morphological, or motoric). The significantly stronger correlation (observed in plot A, Figure 2) between the BP timing of typical and atypical slopes in contralateral legs, compared to the covariance level between BP of nondominant and dominant legs for both thresholds (as shown in panel D of Figure 3 and Figure 4), further bolsters this hypothesis. Regardless of a relatively low number of cases in our study, those results indicate that some bilateral sharing or compensations of asymmetry may be presented in NIRS-based estimates during incremental exercise. However, to our knowledge, no research has been published looking into this kind of effect, and future experimental studies on this topic are needed. 

### 4.2. Agreement between VT and SmO_2_ BP and Effect of Age Group

The main focus of the present study was to analyse the agreement between two different methods for determining AeT and AnT and to control the effect of cyclists’ Age Group (combined effect of age and training history) and performance characteristics to mismatch between systemic and local threshold values. When we analysed the whole sample together, similarly to most previous studies [34], we found relatively strong correlations between the two methods at the group level, and associations were stronger between AnT characteristics compared to AeT measures, also being described by earlier authors [17,34,47]. The group mean values also did not show differences between the two methods for AnT measures like most previous studies [34], but for AeT, the P_kg_ values of BP1 for the DO leg and averaged measures between legs were significantly lower than the corresponding VT1 value, also being reported by Feldmann et al. [17]. When we compared the two methods in separate Age Groups, then no differences were found between VT and any BP-related P_kg_ values, neither in AeT nor AnT measures for Junior cyclists, but a significantly earlier occurrence (at lower P_kg_ values) of AeT at the local (i.e., NIRS) level compared to the systemic response was found in the Seniors group, as well as for DO-leg BP2 for AnT. The between-group differences for said bias values tended to be higher for the Seniors group. However, those differences did not reveal a level of statistical significance, mainly because of relatively heterogeneous VT to BP differences in the Juniors group. 

Most earlier studies have demonstrated no differences between NIRS-based local and systematic AeT measures [34]. At the same time, relatively large methodological variability is encountered. Parameters that could influence the results are as follows: study participants, exercise protocol, NIRS equipment, NIRS measurements (oxygenated (O_2_Hb) or deoxygenated Hb (HHb), tissue saturation index (TSI), or SmO_2_), and systemic threshold (VE or lactate) characteristics, as well as signal processing or BP evaluation methods [34]. At the same time, the tendency to reach the local BP first at the AeT level matches well with the currently known mechanism, which evokes an aerobic threshold [19]. At the aerobic threshold, a local immediate response to load change is the earliest to occur and is followed by an upstream physiological global response to support locally increased demand for substrate deliveries. It is proposed that more glycolytic active type II muscle fibres are activated during a workload increase [48] and therefore, more lactate is produced. At the same time, when the muscle’s capacity to use the lactate energy locally is reached [49], the lactate is leaking out of the muscle. Therefore, buffering systems have to be more active and this results in a higher production of CO_2_, which causes an elevated ventilation rate [38]. It is noted that the majority of increased metabolic demands during a workload increase come from skeletal muscles carrying out mechanical work [50], and knee extensors, including VL, as part of this muscle group, are reported to be the main power producer during submaximal cycling [51]. At the same time, during cycling, almost all leg muscles are activated for pedalling power production [52], but with different contribution levels in different work rates [52,53,54] and the majority of the pelvis region and upper body muscles are used to stabilise cyclists’ position to support propulsive force production, especially during higher workloads [55]. It can be hypothesised that large between-cyclist heterogeneity in muscle groups’ usage/activity patterns [56,57,58,59] and also stability or variability on those patterns on different workloads [56,57,60] and fatigue states [61] play an important role regarding why BP1 in the NIRS signal, according to VT1 positions, diverges for different participants. 

Research evidence has demonstrated that even for the same VL muscle, the NIRS signals show remarkable spatial heterogeneity in reactions to workload change [28]. The reasons why BP1 occurred earlier than VT1 in the Seniors group but not for Juniors could be related to more stable intra- and inter-muscle activity patterns developed during continuous years of cycling [59] for Seniors. Also, more pronounced adaptions in local muscle morphology are evident for long-term endurance training [62]. Years of endurance cycling training have also correlated with a higher proportion of more oxidative type I muscle fibres in VL muscle [63]. Our study results demonstrated significantly more endurance-directed phenotype measures of Senior cyclists than Juniors, being indirect evidence for possibly higher type I muscle fibre prevalence among older cyclists. The other result of the present study, handled in more detail later in the text, demonstrated that, regardless of the Age Group, a more endurance-directed phenotype and higher VO_2_ value at VT2 are related to relatively lower threshold P_kg_ values (earlier occurrence) at the local level compared to the systemic threshold. A similar tendency was also demonstrated between the Seniors and Juniors groups.

Our study results demonstrated that regardless of higher ATT measures and lower aerobic performance characteristics, both related to higher absolute SmO_2_ values [13], in the Seniors group, none of the measured SmO_2_ values (Table 3) were statistically different between Age Groups; reversely, Seniors tended to have lower minimum and maximum SmO_2_ values during the exercise. This can also be interpreted as indirect evidence of more pronounced peripheral adaptation for Senior cyclists. The Seniors group also demonstrated remarkably higher homogeneity, expressed as lower LOA, in differences between local and systemic threshold values. At the same time, our two study groups differed not only by training experience but also by age, and the possible effect of aging on “metabolic inertia” [64] can play a role in elongated latency between local and systemic responses to a workload increase for Seniors. Future studies with participants of a similar age but different training experiences are needed to clarify the effect of training stasis on differences between local and systemic thresholds.

Despite relatively good agreement between threshold methods at the group level, the Bland–Altman analysis indicated a notable lack of agreement on an individual basis, a result that aligns with findings from previous studies [65,66]. The Limits of Agreement (LOA) for bias values were, in most cases, within a range more extensive than 1 W/kg. This value is significantly higher than the one typically used to differentiate cyclists between various performance levels [35]. The LOA tended to be larger between AeT-related VT and BP measures and for the ND leg and smaller for AnT-related measures and averaged values of contralateral legs. The same tendency for better agreement between the systematic physiological response and DO-side SmO_2_ dynamics compared to the ND side and an even better match with the averaged value of contralateral measures have been demonstrated previously [32]. At the same time, no statistical differences were found between ND and DO legs in BP-related power values at the group level, but at the individual level, the LOA between contralateral P_kg_@BP values at both threshold levels tended to be even higher than that found between local and systemic measures. It may indicate that NIRS-derived measures from different body sides add unique information to systemic reactions. Those measurements would be more reasonable to handle as sources of additional information for the systemic response and not as the replacement of methods evaluating global metabolic responses, also being noted by some previous authors [12,65]. It should be noted that sources of bias between two threshold evaluation methods can also arise from different methodological aspects, like validity and reliability of used apparatuses, sensor placement, used exercise protocol and its influence on dynamics of the biological signal, signal processing and filtering methods, differences in BP and VT evaluation methods, and human errors during data collection and manipulation [49]. Some errors in the dynamics of biological signals can be related to random changes in the movement and breathing patterns of studied participants, and this also influences BP and VT computation and evaluation. 

### 4.3. The Effect of Cycling Performance Measures to Agreement between VT and SmO_2_ BP

Regardless of previously described methodological biases, some interindividual variations in the bias between systemic and local thresholds may have a biological origin and can give additional information about athletes’ functional status or phenotype. For this purpose, we studied associations between bias values between two threshold evaluation methods and cyclists’ performance measures. The main biological characteristic that is directly associated with NIRS measurements is ATT, and generally, higher ATT values are related to higher SmO_2_ values at any level of exercise intensity [13]. It is plausible to speculate that the amount of adipose tissue thickness could affect the dynamics of the SmO_2_ signal during incremental exercise. This is because the metabolic activity in subcutaneous adipose tissue is significantly lower compared to muscle tissue. Furthermore, the oxygenated blood flow involved in heat dissipation during an increased work rate might vary among individuals with differing ratios of subcutaneous and muscle tissues within the NIRS measurement range. This speculation was not supported by our study and we did not find any significant correlations (r = 0.135; *p* = 0.45 and 0.124; *p* = 0.49 for Aet and AnT, respectively) between ATT and bias in P_kg_ between two threshold evaluation methods. The low impact of ATT on SmO_2_ signal dynamics may result from relatively low ATT values (≤9 mm for all participants) of our study participants, needed for the correct penetration of NIRS signals in muscle according to methods applied using the Moxy Monitor [33]. Additionally, we did not find any significant correlations between the bias value of the two methods and other measured anthropometrical characteristics (with or without the application of Age Group correction). 

One of the most interesting findings of the present study is that more than one-fourth of inter-subject variability in differences between local and systemic threshold measures can be explained by cyclists’ phenotype measures. It was found that more sprinter-type cyclists, regardless of the Age Group (illustrated in Figure 5, panel B, and Table 7), tend to have reached the systemic threshold more early compared to SmO_2_ signal BP and reversely, athletes who had relatively high P_kg_@VT2 and especially VO_2kg_@VT2 demonstrated lower threshold values for SmO_2_ BP compared to VT. Those relationships were more clearly expressed for AnT measures (Figure 5), but similar tendencies also existed for AeT. It can be hypothesised that sprinter-type cyclists have a larger leg volume, and this may affect NIRS signal readings and dynamics [19]. However, in our study, the cyclists’ phenotype measure did not correlate significantly with any of the measured anthropometric characteristics and therefore, the between-subject quantitative differences in leg muscle volume and ATT were probably not moderators of described relationships. The main muscular level difference between sprint- and endurance-type cyclists is attributed to differences in muscle fibre distribution. Cyclists with better aerobic endurance abilities have proportionally more type I muscle fibres in the VL muscle [63] and probably in other leg muscles. Type I muscle fibres have lower glycolytic power but a higher ability of the mitochondrial power grid to utilise the lactate [49]. It can be speculated that when the balance between lactate production and utilisation is reached at the muscular level, the net lactate leak to central blood circulation is at relatively lower amounts from muscles with a higher proportion of type I muscle fibres than from muscles possessing a relatively higher value of more glycolytic muscle fibres. Also, athletes with higher VO_2_ at submaximal workloads have the potential to utilise centrally leaked lactate “aerobically” in non-locomotor muscles, also being demonstrated in previous NIRS studies [12], and inner organs [49], and through this there can be a delay in the systemic responses, especially VT2, which is more related with the duration of the isocapnic buffering region [49]. Conversely, sprinter-type athletes with higher amounts of glycolytic muscle fibres may have larger lactate leaks from working muscles already in lower work rates, and it can happen from other muscles that were not measured in our study. It is demonstrated that the relative increase in activity of knee extensors stays proportional to the workload increase, but the amount of hamstring muscle group activity increases disproportionally during higher workloads and a high proportion of larger motor units are activated already at lower workloads in the calf muscles [60]. Those factors, associated with higher lactate leaks from other working muscles, not studied in our experiment, in accordance with lower lactate utilisation ability in non-locomotor muscles, may trigger systemic responses more early before the local threshold is reached at VL muscle for more sprinter-type cyclists. At the same time, more scientific evidence is needed to support our speculations about mechanisms behind differences in local and systemic thresholds. 

### 4.4. Future Research and Practical Implications

More working and non-locomotor muscle groups [12] would be needed to study with NIRS methods during different workloads in parallel with systemic response measurements, and exercise protocols with rest periods between more extended workloads [47] would probably be useful to use for threshold evaluation, to separate the effects of the fatigue and work rate. Also, different signal computation methods should be considered for SmO_2_ threshold evaluation [47] to reduce methodological bias arising from signal fluctuation, especially during lower work rates. At the same time, the results of our study demonstrate that wearable NIRS-based devices can add interesting information for monitoring the endurance athlete’s training process, but they cannot be used as a replacement for classical threshold intensity measuring methods. The bilaterally captured NIRS signal can add potentially beneficial scrutiny to local measurements, but more importantly, the comparison of dynamics of contralateral SmO_2_ signals may indicate compensatory mechanisms related to bilateral asymmetries, which also needs future attention in scientific research.

## 5. Limitations

The following methodological limitations of this study must be considered when interpreting the presented results. Notably, the two Age Groups in our study differed not only in age but also in their training histories and performance levels. This overlap prevents a clear distinction between the impact of age and training status on the discrepancies observed between the two threshold evaluation methods.

We used incremental tests with relatively long (3 min) workload durations instead of traditionally used ramp tests. Consequently, the physiological signals likely reached a steady state at lower workloads, potentially influencing the determination of thresholds for both methods. Additionally, maintaining a consistent cycling position during the extended protocol is challenging, and any changes in posture can alter the dynamics of physiological signals, particularly the VL SmO_2_ values. The possible protocol-related bias affects VT1 and BP1 evaluation more, which may also be the reason for lower agreement between AeT measures compared to AnT. 

The aerobic cycling performance characteristics were evaluated during the same incremental test as was used for the threshold detection and this may produce some moderating effect from the test protocol and procedures to the associations between VT2-related characteristics and bias values of the two methods.

## 6. Conclusions

In general, two breakpoints can be defined in the NIRS-captured SmO_2_ signal collected from VL of trained cyclists from different age groups. Nevertheless, regardless of relatively good agreement at the group level with the classically evaluated ventilatory thresholds, the agreement between the two methods at the individual level was too low for interchangeable usage of those methods in the practical training process. Older cyclists with more extended training experience tended to have thresholds first in muscle oxygenation signals compared to in systemic gas exchange responses measured at the mouth; younger, fitter, but less experienced cyclists demonstrated higher heterogeneity in bias values between the two threshold evaluation methods with no average difference between methods. More sprinter-type cyclists tended to have systemic threshold values earlier than local thresholds than athletes with relatively higher aerobic abilities. 

Our study’s findings suggest that simultaneous assessment of both systemic and local threshold values could provide useful insights for profiling cyclists and monitoring their training. Employing NIRS sensors bilaterally can yield more accurate results relative to systemic responses and, crucially, help in identifying potential asymmetries in the musculoskeletal system during regular training. If the use of a single sensor is necessary during cycling tests or training sessions, positioning it on the dominant leg is recommended for optimal results.

## Figures and Tables

**Table 1:** Anthropometric and training-history-related characteristics of study participants grouped as Junior and Senior cyclists.

Variable	Juniors				Seniors			
	N	Mean	SD	CoV	N	Mean	SD	CoV
Age (y)	18	18.2	1.6	0.09	15	43.8	5.2	0.12 *
Height (m)	18	1.847	0.052	0.03	15	1.825	0.052	0.03
Body mass (kg)	18	73.2	7.0	0.10	15	81.5	6.7	0.08 *
BMI (body mass index)	18	21.5	2.0	0.09	15	24.4	1.6	0.07 *
ATT (adipose tissue thickness) (mm)	18	3.9	1.3	0.34	15	6.7	1.7	0.26 *
Cycling stasis (years)	18	5.3	1.3	0.24	15	18.1	6.1	0.34 *
Cycling distance during past season (km)	18	17,061	3607	0.21	15	10,273	2964	0.29 *

*—significant difference between Juniors and Seniors (*p* < 0.05; d > 0.2).

**Table 2:** Characteristics of aerobic and anaerobic performance of Junior and Senior cyclists.

Variable	Group	N	Absolute Values					Relative Values (*/kg)				
			Mean	SD	CoV	*p*	Cohen’s d	Mean	SD	CoV	*p*	Cohen’s d
P@VT1 (W)	Juniors	18	235.0	36.1	0.15	0.032	0.79	3.20	0.31	0.10	<0.001	1.86
	Seniors	15	209.4	27.3	0.13			2.58	0.36	0.14		
P@VT2 (W)	Juniors	18	321.1	40.6	0.13	0.050	0.71	4.39	0.41	0.09	<0.001	1.95
	Seniors	15	293.7	36.0	0.12			3.61	0.39	0.11		
PP (W)	Juniors	18	389.3	41.9	0.11	0.039	0.75	5.33	0.37	0.07	<0.001	2.23
	Seniors	15	358.7	39.0	0.11			4.41	0.46	0.10		
VO_2_@VT1 (mL/min)	Juniors	18	3260	422	0.13	0.326	0.35	44.5	3.2	0.07	<0.001	1.62
	Seniors	15	3120	376	0.12			38.4	4.4	0.11		
VO_2_@VT2 (mL/min)	Juniors	18	4341	495	0.11	0.507	0.24	59.3	4.3	0.07	<0.001	1.60
	Seniors	15	4223	512	0.12			51.9	5.1	0.10		
VO_2_max (mL/min)	Juniors	18	4950	528	0.11	0.892	0.05	67.7	3.9	0.06	<0.001	1.34
	Seniors	15	4924	536	0.11			60.6	6.5	0.11		
Pmax5s (W)	Juniors	18	1236.5	146.0	0.12	<0.001	1.40	16.93	1.66	0.10	<0.001	2.51
	Seniors	15	1011.5	177.1	0.18			12.42	1.96	0.16		
Pmax30s (W)	Juniors	18	912.9	117.9	0.13	0.01	0.96	12.48	1.10	0.09	<0.001	2.46
	Seniors	15	797.9	122.2	0.15			9.78	1.11	0.11		
Phenotype (P5s/P@VT2 (a.u.))	Juniors	18	3.89	0.53	0.14	0.018	0.87					
	Seniors	15	3.45	0.46	0.134							

**Table 3:** Characteristics of minimum, maximum, and BP SmO_2_ values in original scale for Junior and Senior cyclists.

Variable	Leg	Group	N	Mean	SD	Min	Max	CoV	*p*	Cohen’s d
Min SmO_2_ (%)	ND	Juniors	18	12.8	6.4	1.7	26.7	0.50	0.18	0.48
		Seniors	15	9.8	5.8	2.0	23.0	0.59		
	DO	Juniors	18	14.0	8.9	3.0	31.0	0.64	0.12	0.33
		Seniors	15	8.9	4.0	1.3	17.2	0.46		
Max SmO_2_ (%)	ND	Juniors	18	61.1	8.6	46.1	79.7	0.14	0.16	0.51
		Seniors	15	57.2	6.0	50.6	72.9	0.11		
	DO	Juniors	18	61.3	8.9	50.1	80.7	0.15	0.24	0.24
		Seniors	15	56.8	5.5	44.2	66.3	0.10		
BP1 SmO_2_ (%)	ND	Juniors	17	40.2	11.3	18.2	61.7	0.28	0.73	−0.12
		Seniors	15	41.5	10.4	24.5	61.3	0.25		
	DO	Juniors	18	43.2	13.9	21.0	72.3	0.32	0.88	−0.05
		Seniors	15	43.9	10.7	25.9	63.0	0.24		
BP2 SmO_2_ (%)	ND	Juniors	18	19.0	9.1	3.6	41.4	0.48	0.94	0.03
		Seniors	15	18.7	9.4	5.5	34.8	0.50		
	DO	Juniors	18	21.3	11.5	4.7	48.8	0.54	0.26	0.40
		Seniors	15	16.9	10.1	2.4	39.1	0.60		

**Table 4:** Characteristics of relative power values at SmO_2_ *breakpoints* for Junior and Senior Cyclists.

Variable	Group	N	Mean	SD	CoV	*p*	Cohen’s d
P_kg_@BP1 of ND SmO_2_ (W/kg)	Juniors	17	3.20	0.50	0.16	<0.001	1.82
	Seniors	15	2.41	0.35	0.14		
P_kg_@BP1 of DO SmO_2_ (W/kg)	Juniors	18	3.13	0.41	0.13	<0.001	1.92
	Seniors	15	2.34	0.41	0.18		
P_kg_@BP1 of Avr SmO_2_ (W/kg)	Juniors	18	3.16	0.39	0.12	<0.001	2.11
	Seniors	15	2.38	0.35	0.15		
P_kg_@BP2 of ND SmO_2_ (W/kg)	Juniors	18	4.36	0.42	0.10	<0.001	1.68
	Seniors	15	3.65	0.42	0.12		
P_kg_@BP2 of DO SmO_2_ (W/kg)	Juniors	18	4.41	0.44	0.10	<0.001	2.17
	Seniors	15	3.47	0.43	0.13		
P_kg_@BP2 of Avr SmO_2_ (W/kg)	Juniors	18	4.39	0.38	0.09	<0.001	2.13
	Seniors	15	3.56	0.40	0.11		

**Table 5:** Differences between relative power values at VT1 and BP1 according to Age Groups.

	VT1—BP1 Power Difference (W/kg)							
Variable	Group	N	Mean	SD	Limits of Agreement			Cohen’s d
					Lower	Upper	*p*	
VT1—BP1 of ND SmO_2_	Juniors	17	−0.01	0.45	−0.88	0.87	0.195	−0.47
	Seniors	15	0.17	0.25 *	−0.32	0.65		
VT1—BP1 of DO SmO_2_	Juniors	18	0.07	0.34	−0.60	0.74	0.127	−0.55
	Seniors	15	0.24	0.28 *	−0.30	0.79		
VT1—Avr BP1 of SmO_2_	Juniors	18	0.04	0.32	−0.59	0.68	0.103	−0.59
	Seniors	15	0.21	0.22 *	−0.22	0.63		

*—significant intra-group difference between P_kg_@VT1 and P_kg_@BP1 values (*p* < 0.05).

**Table 6:** Differences between power values at VT2 and BP2 according to Age Groups.

	VT2—BP2 Power Difference (W/kg)							
Variable	Group	N	Mean	SD	Limits of Agreement			Cohen’s d
					Lower	Upper	*p*	
VT2—BP2 of ND SmO_2_	Juniors	18	0.03	0.30	−0.55	0.62	0.503	0.24
	Seniors	15	−0.04	0.31	−0.66	0.58		
VT2—BP2 of DO SmO_2_	Juniors	18	−0.02	0.31	−0.63	0.59	0.096	−0.60
	Seniors	15	0.14	0.19 *	−0.23	0.51		
VT2—Avr BP2 of SmO_2_	Juniors	18	0.01	0.23	−0.45	0.46	0.58	−0.20
	Seniors	15	0.05	0.20	−0.35	0.45		

*—significant intra-group difference between VT2 and BP2 values (*p* < 0.05).

**Table 7:** Correlations between cyclists’ performance characteristics and the bias between relative power values of Ventilatory thresholds and SmO_2_ breakpoints for all samples and for separate Age Groups.

Variable	VT1-Avr BP1 Power Difference (W/kg)			VT2-Avr BP2 Power Difference (W/kg)		
	Juniors (n = 17)	Seniors (n = 15)	All # (n = 32)	Juniors (n = 18)	Seniors (n = 15)	All # (n = 33)
P_kg_@VT1 (W/kg)	0.263	0.335	0.288	0.268	−0.022	0.136
P_kg_@VT2 (W/kg)	0.084	0.423	0.219	0.388	0.249	0.331
PP_kg_ (W/kg)	0.09	0.366	0.208	0.184	0.103	0.143
VO_2kg_@VT1 (mL/min/kg)	0.079	0.291	0.172	0.062	0.18	0.117
VO_2kg_@VT2 (mL/min/kg)	0.12	0.41	0.249	0.567 *	0.409	0.488 **
VO_2kg_max (mL/min/kg)	0.08	0.455	0.258	0.429	0.227	0.303
P_kg_max5s (W/kg)	−0.293	−0.238	−0.264	−0.342	−0.295	−0.317
P_kg_max30s (W/kg)	−0.06	0.059	−0.022	−0.215	0.088	−0.088
Phenotype (P5s/P@VT2 (a.u.))	−0.273	−0.575 *	−0.385 *	−0.508 *	−0.526 *	−0.515 **

* *p* < 0.05, ** *p* < 0.01; # partial correlation with controlling variable: Age Group. One Junior cyclist was removed from analysis of AeT measures as an outlier.

## Data Availability

The original data file is available as a Supplementary Table (File S1).

## Supplementary Materials

The following supporting information can be downloaded at: https://www.mdpi.com/article/10.3390/sports12020040/s1.
